# Diagnostic accuracy of different body weight and height-based definitions of childhood obesity in identifying overfat among Chinese children and adolescents: a cross-sectional study

**DOI:** 10.1186/s12889-015-2152-0

**Published:** 2015-08-20

**Authors:** Lin Wang, Stanley Sai-chuen Hui

**Affiliations:** Department of Sports Rehabilitation, Shanghai University of Sport, Shanghai, China; Department of Sports Science and Physical Education, The Chinese University of Hong Kong, Hong Kong, China

## Abstract

**Background:**

Various body weight and height-based references are used to define obese children and adolescents. However, no study investigating the diagnostic accuracies of the definitions of obesity and overweight in Hong Kong Chinese children and adolescents has been conducted. The current study aims to investigate the diagnostic accuracy of BMI-based definitions and 1993 HK reference in screening excess body fat among Hong Kong Chinese children and adolescents.

**Methods:**

A total of 2,134 participants (1,135 boys and 999 girls) were recruited from local schools. The foot-to-foot BIA scale was applied to assess %BF using standard methods. The criterion of childhood obesity (i.e., overfat) was defined as over 25 %BF for boys and over 30 %BF for girls. Childhood obesity was also determined from four BMI-based references and the 1993 HK reference. The diagnostic accuracy of these existing definitions for childhood obesity in screening excess body fat was evaluated using diagnostic indices.

**Results:**

Overall, %BF was significantly correlated with anthropometry measurements in both genders (in boys, *r* = 0.747 for BMI 0.766 for PWH; in girls, *r* = 0.930 for BMI 0.851 for PWH). The prevalence rates of overweight and obesity determined by BMI-based references were similar with the prevalence rates of obesity in the 1993 HK reference in both genders. All definitions for childhood obesity showed low sensitivity (in boys, 0.325–0.761; in girls, 0.128–0.588) in detecting overfat. Specificities were high for cut-offs among all definitions for childhood obesity (in boys, 0.862–0.980; in girls, 0.973–0.998).

**Conclusions:**

In conclusion, prevalence rates of childhood obesity or overweight varied widely according to the diagnostic references applied. The diagnostic performance for weight and height-based references for obesity is poorer than expected for both genders among Hong Kong Chinese children and adolescents. In order to improve the diagnosis accuracy of childhood obesity, either cut-off values of body weight and height-based definitions of childhood obesity should be revised to increase the sensitivity or the possibility of using other indirect methods of estimating the %BF should be explored.

## Background

In large-scale population surveys and public health screening, anthropometry indices [i.e., body mass index (BMI), weight-for-height, waist circumference] are commonly used as surrogates for body fat [1, 2]. Various definitions of childhood obesity have been developed using these anthropometry measures. BMI is one of the most highly recommended and widely used tools for defining childhood obesity [3–5]. Three commonly used tools are World Health Organization (WHO) BMI-based [6], International Obesity Task Force (IOTF) BMI-based [7], and Centers for Disease Control and Prevention (CDC) BMI-based references [8]. In 2005, the Working Group on Obesity in China (WGOC) developed a BMI-based definition for childhood obesity in Chinese school-aged children [9]. Through these definitions, the cut-off points for childhood obesity are determined using a statistical approach, where an arbitrarily chosen value from a reference distribution defines childhood obesity [7, 10, 11]. The relationship between BMI, body fat percentage (%BF), and health risk is not well established for children and adolescents, including those in Hong Kong.

Relative weight-for-height is used to define childhood obesity in some countries, especially in Asia [12]. In this approach, cut-off points for being obese is defined as a set percent over a standard weight for a given height in the individual’s age and sex population. The standard weight is usually determined as the mean or median determined from a normal reference distribution for the population, and the most commonly used cut-off point for obesity is 110 % or 120 % standard weight-for-height [13]. For the past 20 years in Hong Kong, childhood obesity has been defined as a body weight of over 120 % of the median weight for height according to the local reference (1993 HK reference) [14], where reference values for height are available only for up to 175 cm in boys and 165 cm in girls. Overweight is not defined in the standard.

At the population level, %BF is most commonly evaluated using BMI. However, international studies have found that BMI cut-offs do not provide an equivalent measurement of %BF and are associated with health risks across different ethnic groups [15]. One possible reason to explain this result is the differences in body composition due to ethnicity. Compared with their African-American and European counterparts, Asian children and adolescents have higher %BF at the same BMI level [16–18], raising the possibility of underestimating excess body fat in Asian groups when using BMI as indirect measure. Such situation is undesirable because it will influence the access of public health services of those who most need intervention. Therefore, large-scale studies need to determine the relationship between anthropometry measurements and %BF, including Hong Kong Chinese children and adolescents. The purposes of the current study was to investigate the diagnostic accuracy of BMI-based definitions and 1993 HK reference in screening excess body fat among Hong Kong Chinese Children and adolescents.

## Methods

### Participants

The study was conducted among 2,191 Chinese children and adolescents aged 9–19 years, representing a convenience sample enrolled in Hong Kong primary and secondary schools. After excluding 57 participants with missing measurement data, 2134 participants (1135 boys and 999 girls) were included in the data analysis. All participants self-identified as ethnic Chinese. Written informed consents were obtained from all participants and their parents. The current study was approved by the Joint Chinese University of Hong Kong-New Territories East Cluster Clinical Research Ethics Committee.

### Anthropometry measurement

Body weight and height were measured with minimal clothing and bare feet. Body height was measured to the nearest 0.5 cm using a portable stadiometer (Seca 213 portable stadiometer, Seca GmbH & Co. Kg, Hamburg, Germany). Body weight was measured to the nearest 0.1 kg using a standard scale (Tanita TBF 410, Tanita, Tokyo, Japan). BMI was calculated as body weight in kilograms divided by body height in meter squared.

### Body fat measurement

Body fat percentage (%BF) was measured by a foot-foot bioelectric impedance analyzer (BIA, Tanita TBF 410, Tanita, Tokyo, Japan). The analyzer incorporates both a weighing scale and a foot-foot bioelectric impedance analyzer. BIA measurements were taken at least 2 h after a meal and on an empty bladder. Age, gender, and height were manually entered. Dry tissue papers were used to clean the soles of participants, who were asked to dress in light sportswear and stand on the four metal electrodes. The device displayed body weight and %BF predicted using embedded equations. Studies showed high correlation between %BF estimated by dual energy X-ray absorptiometry (DXA) and that estimated by Tanita foot-to-foot BIA scales [19, 20]. Tanita foot-to-foot BIA scales are portable devices that perform well in measuring %BF among Chinese Children [20].

### Definition of excess fatness

Generally, obesity is defined as an abnormal or excessive fat accumulation that presents health risks to children and adolescents [21]. In current study, excess body fat was defined as over 25 % body fat for boys and over 30 % for girls [22]. The cut-off was equivalent to overweight or obesity in accordance with definitions for childhood overweight or obesity in current study. The cut-offs are strongly associated with cardiovascular risk factors in children and adolescents, and are therefore widely used excess body fat definitions [23–25].

### Classification of participants

For the analysis, participants were classified according to BMI and local weight-for-height, definitions for childhood obesity. For overweight and obese classifications, four BMI-based definitions were used: IOTF BMI-based [7]; CDC 2000 BMI-based [8]; WHO BMI-based [6]; and and WGOC BMI-based references [9]. The present study also used the 1993 HK reference (i.e., childhood obesity is defined as having a body weight of over 120 % of the median weight for height in the 1993 growth curve) to define childhood obesity [14]. In the growth curve, reference values are only available for heights of up to 175 cm in boys and 165 cm in girls. Being overweight is not defined in the standard.

### Data reduction and statistical analysis

For data analysis, participants were categorized into subgroups according to gender and age. Data were represented as mean and standard deviation (SD). Statistical analysis was performed using SPSS 17.0 statistical software (SPSS Inc. Chicago, IL, USA).

Between–Gender comparisons in anthropometry measurement and %BF were performed using Independent t-tests. After adjusting for age, the association between anthropometry measurements and %BF were assessed using partial correlation coefficients. Chi-square tests were used to determine the differences in the prevalence of childhood overweight and obesity.

The diagnostic accuracy of the different classifications in detecting excess body fat were evaluated by comparing their respective diagnostic accuracy indices, including sensitivity (SE), specificity (SP), positive predictive values (PPV), likelihood ratio for positive test results (LR+), and Youden’s index (YI). SE (true positive rate) is the proportion of truly obese/overweight subjects correctly identified by classification. SP (true negative rate) is the proportion of truly non-obese/non-overweight subjects correctly identified by classification. The PPV of a screening method is the proportion of being truly obese/overweight when a participant is classified as obese/overweight by a screening method. LR+ is the ratio of true positive rate (SE) to false positive rate (1-SP), and is the best indicator for ruling-in diagnosis; a higher LR+ indicated that the test was more indicative of the disease. Youden’s index is an overall measure to summarize SE and SP, and calculated using the formula: YI = (SE + SP)-1. Chi-square tests were used to determine the differences in the sensitivity and specificity between genders.

## Results

### Characteristics of participants

Table 1 presents the physical characteristics of current participants. The study population consisted of 2,134 children and adolescents aged 9–19 years with 53.2 % boys (age, 13.7 ± 2.9 y) and 46.8 % girls (age, 13.9 ± 2.7 y). For the total samples, boys had higher body height and heavier body weight than girls. Despite the larger BMI of boys, their %BF was significantly lower than girls.

**Table 1:** Descriptive statistics of physical characteristics of participants

	Boys (*n* = 1135)			Girls (*n* = 999)		
	Mean ± SD	5th percentile	95th percentile	Mean ± SD	5th percentile	95th percentile
Age (year)	13.7 ± 2.9	9.4	18.4	13.9 ± 2.7	9.4	17.8
Body height (cm)	159.6 ± 14.5	133.0	179.0	152.7 ± 10.0	132.0	166.0
Body weight (kg)	53.4 ± 15.5	29.5	80.5	46.9 ± 11.9	28.7	66.9
BMI (kg/m^2^)	20.6 ± 4.0	15.3	28.5	19.9 ± 3.7	15.1	26.6
%BF (%)	19.9 ± 8.8	8.8	37.0	28.1 ± 7.1	18.4	41.1

### Age-adjusted correlation among the different indices of obesity

After adjusting for the participants’ age, BMI and PWH correlated well with each other in both genders (in boys, *r* = 0.977, *p* < 0.001; in girls, *r* = 0.901, *p* < 0.001). Overall, %BF was significantly correlated with anthropometry measurements in both genders. In boys, the correlation coefficients between %BF and anthropometry measurements were 0.747 for BMI and 0.766 for PWH (*p* < 0.001). In girls, the correlation coefficients were 0.930 for BMI and 0.851 for PWH, (*p* < 0.001). Compared with their male counterparts, girls had better correlation between BMI, PWH and % BF.

### Prevalence rates of overweight/obesity

The prevalence of overfat as estimated from the %BF was 24.4 % [95 % confidence interval (CI) = 21.9-26.9 %] in boys, and 34.5 % (95 % CI = 31.6-37.4 %) in girls. Girls have higher prevalence rate of overfat than boys (*X*^2^ = 26.40, *p* < 0.001). Body heights in 12.0 % boys exceeded 175 cm, and those in 6.1 % girls exceeded 165 cm, indicating that these participants were not categorized with body weight status using the 1993 HK reference. The prevalence of obesity/overweight varies by the definitions used. The 1993 HK reference obesity rates were higher, but similar when OW and OB rates are combined in all BMI-based references (Table 2).

**Table 2:** Prevalence rates of obesity and overweight estimated by existing definitions for childhood overweight and obesity

	%BF	HK 1993	BMI based definitions							
			IOTF-BMI		CDC-BMI		WHO-BMI		WGOC-BMI	
	Overfat	OB	OB	OB & OW	OB	OB & OW	OB	OB & OW	OB	OB & OW
Boys (*n* = 1135)	24.4 % (21.9-26.9)	29.2 % (26.4-32.0) (*n* = 999) ^a^	8.5 % (6.9-10.1)	28.0 % (25.4-30.6)	12.4 % (10.5-14.3)	27.4 % (24.8-30.0)	14.5 % (12.5-16.6)	33.4 % (30.7-36.1)	13.3 % (11.3-15.3)	30.5 % (27.8-33.2)
Girls (*n* = 999)	34.5 % (31.6-37.4)	21.5 % (18.9-24.1) (*n* = 938) ^a^	4.4 % (3.1-5.7)	20.0 % (17.5-22.5)	6.6 % (5.1-8.1)	19.2 % (16.8-21.6)	6.9 % (5.3-8.5)	22.3 % (19.7-24.5)	9.7 % (7.9-11.5)	21.8 % (19.2-24.4)

All data are presented as mean (95 % confidential interval, 95%CI)

*OB* obesity, *OB & OW* overweight and obesity, *BMI* body mass index, *%BF* body fat percentage measuring by BIA, *HK 1993* 1993 Hong Kong reference, *IOTF-BMI* International Obesity Task Force BMI reference, *CDC-BMI US* Centers for Disease Control and Prevention BMI reference, *WHO-BMI* World Health Organization reference*, WGOC-BMI* Working Group on Obesity in China BMI reference

^a^Some subjects are not included in the analysis for their higher body height

### Sensitivity and specificity

1. Sensitivity and specificity of references for obesity

As shown in Table 3, the sensitivity of the references for obesity varied from 0.325 to 0.761 in boys and 0.128 to 0.588 in girls. The 1993 HK reference had a higher sensitivity compared with all BMI-base references for obesity in both genders. Overall, all BMI-based references showed a very low sensitivity (0.128–0.278) in detecting excess body fat among girls. The results indicated that about 68–87 % obese girls are mislabeled as non-obese according to BMI-based references for obesity. Moreover, the Chi-square test found that the rate of sensitivity for each reference for obesity was higher among boys than that among girls.

**Table 3:** Diagnostic indices of different definitions for childhood obesity/overweight in screening excess %BF

	References	SE		SP		YI		PPV		LR+	
		Boys	Girls	Boys	Girls	Boys	Girls	Boys	Girls	Boys	Girls
OB	HK-1993	0.761 (188/247)	0.588 (187/318)	0.862 (648/752)	0.973 (603/620)	0.623	0.561	0.644	0.917	5.504	21.447
	WHO-BMI	0.502 (139/277)	0.200 (69/345)	0.971 (832/858)	0.998 (653/654)	0.473	0.199	0.848	0.986	17.201	130.800
	CDC-BMI	0.448 (124/277)	0.191 (66/345)	0.980 (840/858)	0.998 (653/654)	0.428	0.190	0.978	0.985	22.567	125.113
	IOTF-BMI	0.325 (90/277)	0.128 (44/345)	0.993 (851/858)	0.998 (653/654)	0.318	0.126	0.938	0.978	46.408	83.409
	WGOC-BMI	0.473 (131/277)	0.278 (96/345)	0.977 (837/858)	0.998 (653/654)	0.450	0.277	0.868	0.990	20.265	181.900
OW & OB	WHO-BMI	0.845 (234/277)	0.542 (187/345)	0.856 (734/858)	0.945 (618/654)	0.678	0.487	0.619	0.839	5.027	9.847
	CDC-BMI	0.762 (211/277)	0.493 (170/345)	0.883 (757/858)	0.966 (632/654)	0.645	0.459	0.670	0.885	6.528	14.648
	IOTF-BMI	0.765 (212/277)	0.504 (174/345)	0.878 (752/858)	0.961 (628/654)	0.643	0.464	0.669	0.870	6.247	12.686
	WGOC-BMI	0.801 (222/277)	0.548 (189/345)	0.855 (733/858)	0.956 (625/654)	0.657	0.503	0.642	0.867	5.539	12.354

*OB* obesity, *OB & OW* overweight and obesity, *HK 1993* 1993 Hong Kong reference, *IOTF-BMI* International Obesity Task Force BMI reference, *CDC-BMI* US Centers for Disease Control and Prevention BMI reference, *WHO-BMI* World Health Organization reference, *WGOC-BMI* Working Group on Obesity in China BMI reference, *SE* sensitivity, *SP* specificity, *YI* Youden’s index, *PPV* positive predictive values, *LR+* likelihood ratio for positive test results

All references examined in the study had very high specificity (0.973–0.998) in girls. Among boys, all BMI-based references showed higher specificity (>0.950) than the 1993 HK reference (0.862). YI indicated that the 1993 HK reference performed better than BMI-based references in both genders (YI = 0.623 for boys; 0.561 for girls). Moreover, results of PPVp (0.848–0.990) and the LR+ (17.201–181.900) indicated that all BMI-based references for obesity and the 1993 HK reference for obesity excluding among boys, performed well to assess subjects in both genders with excess fat. The values of PPV and LR+ among boys using the 1993 HK reference were 0.664 and 5.504, respectively. For the WGOC-BMI reference, obesity among boys was defined by the PPV value of 0.868, where the PPV value means that 86.8 % of the subjects is truly obese as per the assessment by WGOC-BMI for obesity. The LR+ value at 20.265 indicated that subjects with %BF > 25 % are 20.265 times as likely as subjects with %BF < 25 % to be categorized as obese.

2. Sensitivity and specificity of references for overweight and obesity

Table 3 presents values of diagnostic accuracy indices of BMI-based references for overweight and obesity in assessing excess body fat. Among boys, the sensitivity values of the references varied from 0.762 to 0.845. Among girls, the rates varied from 0.493 to 0.548. These findings indicate that overweight and obese boys can reach up to 23.8 %, while overweight and obese girls can reach up to 51.7 % who are misclassified into the normal weight group. Moreover, the Chi-square test found that the rate of sensitivity for each reference for overweight and obesity was higher among boys than among girls.

All BMI-based references for overweight and obesity showed high specificity rates in both genders. The rates varied from 0.855 to 0.883 among boys, and from 0.945 to 0.966 among girls. Similar YI were found in BMI-based references for overweight and obesity among boys (0.630–0.697) and among girls (0.459–0.503). PPV varied from 0.619 to 0.670 among boys, and from 0.839 to 0.870 among girls. LR+ values were 5.027–6.528 among boys, while 9.847–14.648 among girls.

## Discussion

The classification of childhood obesity is complicated. The diagnostic performance of simple and low-cost anthropometry-based indices in screening excess body fat is of practical importance in public health surveys and in clinical evaluations for the health practitioner. Various anthropometry index definitions were developed to define childhood obesity [25, 26]. In the current study, we validated BMI and PWH as predictors of excess fat with the use of %BF evaluation by BIA as the criterion. More than 2000 Hong Kong Chinese children and adolescents aged 9 to 19 years old were recruited for the study. The results of the study suggest that 1) local PWH-based references for obesity had higher sensitivity than BMI-based references for obesity; 2) none of BMI-based references for obesity achieved optimal rates of sensitivity.

Ko et al. investigated the prevalence of obesity among Hong Kong adolescents using four diagnostic references: the 1993 HK reference, the IOTF-BMI reference, the CDC-BMI reference, and the WGOC-BMI reference [27]. The 1993 HK reference gave an exceedingly high figure for obesity compared with BMI-based references [27]. Yeung and Hui observed that the prevalence of childhood obesity in Hong Kong for 2006 was 22.67 % and 16.88 % for boys and girls, respectively, using the 1993 HK reference. When the cut-offs determined by IOTF were used, the prevalence rates of childhood obesity for boys and girls were 6.33 % and 3.08 %, respectively [28]. Moreover, the prevalence rates of overweight using the cut-offs determined by IOTF were similar to those for obesity using the 1993 HK reference [28]. In the current study, the 1993 HK reference obesity rate was higher, but similar when OW and OB rates are combined in all BMI-based references. The results are consistent with the findings of previous studies. Moreover, in the 1993 HK reference, reference values for height are available only for up to 175 cm among boys and 165 cm among girls. So et al. found that the body height of Hong Kong children and adolescents aged 6–18 years increased significantly from 1993 to 2005/2006 for both genders [29]. In the current study, we found that 12.0 % boys and 6.1 % girls were not evaluated by the 1993 HK reference. Thus, the 1993 HK reference for obesity should be adjusted to cover populations with excessive body height. Moreover, the IOTF-BMI reference may underestimate or overestimate obesity in comparison to national definitions [30]. Among Asian children and adolescents, the IOTF-BMI reference for obesity gave a lower rate for childhood obesity than those by other BMI references [31]. Similar findings are shown by the current study. IOTF-BMI reference has lower obesity rate than those by other BMI based references in both genders (Table 2), the difference may be attributed by the different reference population, smoothing methods and approached methods to BMI cut-offs of different obesity references.

In the current study, BMI and PWH showed strong positive correlations with %BF which was measured by BIA in both genders. The results were consistent with the findings in previous studies [32–35]. Despite the use of correlation coefficients to estimate the degree of closeness of the linear relationship between variables, correlation coefficients cannot detect diagnostic performance of anthropometry-based indices in screening excess body fat.

Among the BMI-based references for obesity (IOTF, WHO, CDC, and WGOC) analyzed in the current study, none of the references achieved optimal rates of sensitivity (0.325–0.502 for boys and 0.128–0.278 for girls). However, specificities were high (>0.9) for both genders. The result is consistent with the finding of previous studies [25, 31–34, 36]. However, large discrepancies between sensitivity and specificity rates in different studies were found. The differences in sensitivity and specificity rates may be explained by the reference population, adopted cut-offs from various references, methods used to assess %BF, and the cut-offs for excess fat [31, 33].

In previous studies, researchers found that local PWH-based references for obesity had higher sensitivity than BMI-based references for obesity [32, 36]. A similar finding was also evident in the current study. The result suggests that the 1993 HK reference may currently better reflect the true prevalence of obesity among Hong Kong children and adolescents. In addition, when BMI-based references for overweight and obesity were used to assess subjects with excess fat, higher sensitivities and lower specificities were found in both genders in comparison with those of BMI-based reference for obesity. Similar results were reported by other studies [25, 36, 37]. As shown in Table 3, we observed similar sensitivity and specificity rates in screening excess body fat between the 1993 HK reference for obesity and BMI-based references for overweight and obesity. The rates for overweight and obesity determined by the BMI-based references were similar with the rate for obesity by the 1993 HK reference for both genders. Thus, BMI cut-offs for obesity should be decreased to accurately estimate excess body fat among Hong Kong Chinese children and adolescents.

In addition, the current study found significant differences in sensitivity between genders. For each reference for obesity and for each reference for overweight and obesity, boys have higher sensitivity than girls. Moreover, girls had lower diagnostic agreements between %BF reference for excess fat and all BMI-based and WC-based references than boys. Similar results have been found by previous studies [25, 31, 35, 37]. However, inconsistent results were found by other studies [33, 36, 38]. As mentioned above, complex factors, such as ethnicity, age ranges of the population, different cut-offs used to define excess fat, and the method in assessing %BF, may influence the diagnostic performance of different references in screening excess %BF.

In general, the 1993 HK reference for obesity is superior compared with BMI-based references for obesity in screening excess %BF among children and adolescents. Moreover, using the cut-offs for overweight, the diagnostic performance of BMI-based references in screening excess body fat can be improved. WC-based references for overweight and obesity may be used with local reference data similar with the 1993 HK reference and the BMI-based references for overweight and obesity. However, among girls, diagnostic performances of BMI-based references, WC references, and the 1993 HK reference are poorer than expected, i.e., lower sensitivity with poorer diagnostic agreement. Therefore, we attempted to identify optimal cut-offs of different anthropometry-based indices in screening excess %BF for both genders.

The current study has several limitations. One of the limitations in the present study is the difference in definitions for age groups, which may influence diagnostic accuracy of different definitions for childhood obesity in screening overfat. We did not factor in the stage of sexual maturation in our assessment of %BF, and the factor may increase possibilities of over- and under-estimation of overweight and obesity among children and adolescents. Moreover, although we used health-related cut-offs to define excess fat [24], the %BF cut-offs have raised criticism on their reference population, sample size, as well as selected health outcomes and risk measures [24]. To evaluate these cut-offs, others health-related criteria, such as blood pressure, blood cholesterol and blood sugar, may be taken into consideration among Hong Kong Chinese children and adolescents for further study. The choice of same %BF cut-off to define overweight or obesity considered as another limitation of this study. The purpose of the study of using this cut-off in the paper is for comparison among different weight and height-based classification criteria. Ideally, overweight and obesity should be defined as different %BF cut-offs. %BF cut-off to define overweight or obesity may increases the practicality of applying of these weight and height-based classification criteria in real world setting. To our knowledge, there is no unambiguous prior statement of %BF cut-offs to define overweight or obesity. Therefore, further studies should be conducted to identify %BF cut-offs to define overweight or obesity using others health-related criteria. In addition, our sample was not a nationally represented sample of Hong Kong children and adolescents. Further studies are necessary to be conducted using a nationally represented sample to confirm the results of the study. Another limitation is the nature of the criteria measures employed in this study. Previous studies found that foot-to-foot BIA scales give a lower mean and higher intra-individual variation in %BF than criteria measure, such as dual-energy X-ray absorptiometry (DXA), hydrostatic weighing [39, 40]. The method is appropriate for estimating %BF in population rather than individual [39, 40]. Foot-to-foot BIA scales may under- or overestimate %BF depending on the size and gender of individual being measured, therefore, some participants in current study may misclassify as “normal” or “overfat”.

## Conclusion

In conclusion, prevalence rates of childhood obesity or overweight varied widely according to the diagnostic references applied. The diagnostic performance for weight and height-based references for obesity is poorer than expected for both genders among Hong Kong Chinese children and adolescents. In order to improve the diagnosis accuracy of childhood obesity, either cut-off values of body weight and height-based definitions of childhood obesity should be revised to increase the sensitivity or should be targeted toward defining local population and ethnic-specific cut-offs for childhood obesity using health-related excess body fat.
